# Swimming training prevents obesity installation and normalizes hypothalamic expressions of GLP1 and leptin receptors in adult offspring born in small litters

**DOI:** 10.31744/einstein_journal/2024AO0619

**Published:** 2024-08-28

**Authors:** Stefani Valeria Fischer, Bruna Schumaker Siqueira, Claudia Regina Capriglioni Cancian, Elisangela Gueiber Montes, Viviane Nogaroto Vicari, Paulo Vinicius Svidnicki, Sabrina Grassiolli

**Affiliations:** 1 Department of General Biology Universidade Estadual de Ponta Grossa Ponta Grossa PR Brazil Department of General Biology, Universidade Estadual de Ponta Grossa, Ponta Grossa, PR, Brazil.; 2 Postgraduate Program in Biosciences and Health Department of Center for Biological and Health Sciences Universidade Estadual do Oeste do Paraná Cascavel PR Brazil Postgraduate Program in Biosciences and Health, Department of Center for Biological and Health Sciences, Universidade Estadual do Oeste do Paraná, Cascavel, PR, Brazil.

**Keywords:** Hypothalamus, Exercise, Metabolic reprogramming, Swimming, Obesity

## Abstract

**Objective:**

Glucagon-like peptide-1 (GLP1) and leptin (Lep) are afferent signals that regulate energy metabolism. Lactational hypernutrition results in hyperphagia and adiposity in adult life, and these events can be prevented by exercise. We evaluated the effects of swimming training on hypothalamic (GLP1-R) and Lep receptor (Lep-R) gene expressions in lactational hypernutrition-induced obesity.

**Methods:**

On the 3^rd^ postnatal day, the litter sizes of lactating dams were adjusted to small litters (SL; 3 pups/dams) or normal litters (NL; 9 pups/dams). After weaning (21 days), NL and SL male rats were randomly distributed to sedentary (Sed) and exercised (Exe) groups. Exercised mice swam (30 min/3 times/week) for 68 days. Food intake and body weight gain were registered. At 92 days, intraperitoneal glucose and insulin tolerance tests were performed and rats were euthanized at 93 days; adipose tissue depots were weighed, and blood counts and plasma biochemical analyses performed. Hypothalamus were isolated to evaluate Lep-R and GLP1-R gene expressions.

**Results:**

Small litters sedentary rats presented increased body weight gain, adiposity, insulin sensibility and higher fasting values of glucose and triglycerides, besides higher hypothalamic gene expressions of Lep-R and GLP1-R, compared to NL_Sed_ animals. SL_Exe_ rats did not develop obesity or metabolic abnormalities and Lep-R and GLP1-R hypothalamic gene expressions were normalized.

**Conclusion:**

Lactational hypernutrition induces obesity and metabolic dysfunction in adult life, in association with higher hypothalamic expressions of the Lep-R and GLP1-R genes. Exercise prevented obesity and improved metabolic state in SL overnourished rats, and normalized their hypothalamic Lep-R and GLP1-R gene expressions.

## In Brief

Lactation is an important period of metabolic programming. Litter reduction, a model of lactational hypernutrition, promotes obesity and metabolic dysfunctions in adulthood. Here, we demonstrate that swim training introduced early in life prevents obesity and metabolic abnormalities in small-litter rats, in part by adjusting the hypothalamic expression of GLP1-R and Lep-R.

## Highlights

Small-litter promotes greater body weight gain, adiposity, and metabolic abnormalities in adulthood.

Small-litter -adult obese rats showed augmented hypothalamic expression of GLP1-R and Lep-R.

Swimming training throughout life avoids obesity and metabolic disorders in small-litter-adult rats.

Regular exercise can normalize GLP1-R and Lep-R hypothalamic expressions in small-litter-adult rats.

Article extracted from author Stefani Valeria Fischer master’s thesis presented to the Postgraduate Program in Evolutionary Biology at the *Universidade Estadual de Ponta Grossa, Ponta Grossa*, PR, Brazil, in partnership with the *Universidade Estadual Oeste do Paraná, Cascavel*, PR, Brazil.

## Graphical abstract

**Figure:**
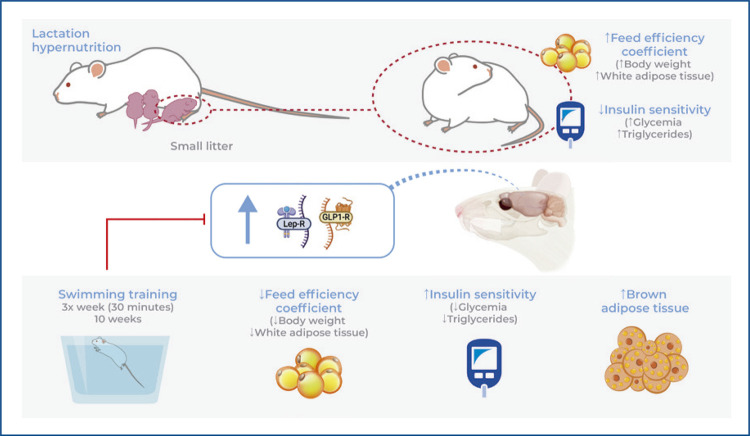

## INTRODUCTION

Nutritional experiences during early developmental periods of pregnancy and lactation may contribute to the onset of metabolic diseases later in life, including obesity.^(1)^ Excessive nutrients at early stages of life can modify gene expression via epigenetic mechanisms, in turn modulating the construction of adaptive responses in tissues involved in energy metabolism and weight control. In this regard, white adipose tissue (WAT), endocrine pancreas, hypothalamus and the intestines are especially plastic during childhood^(2,3)^ and highly influenced by nutritional elements.^(4)^

Several studies in rodents and humans have shown that excessive energy supply during pregnancy and/or the lactation period favors high WAT accumulation throughout life.^(5)^ Moreover, the adult-obesity induced by gestational or lactational high-calorie diets also favors insulin resistance (IR), dyslipidemia and cardiovascular disease at a younger age.^(6,7)^

The hypothalamus is an important neuronal area that controls energy homeostasis^(8)^ and disruptions in neurotransmission in several hypothalamic nuclei can cause altered regulation of body weight and metabolism.^(9)^ The hypothalamic neuronal circuits for controlling body weight and satiety begin to establish early in life^(10,11)^ and are very vulnerable to nutritional insults that can program metabolic changes along life.^(12)^

The control of hypothalamic energy homeostasis is finely regulated by peripheral hormonal signals, which are primarily originated from the WAT and the intestine.^(8)^Accordingly, circulating levels of leptin (Lep) reflect the energy storage in WAT.^(10)^ A high adipocyte triglyceride content results in high Lep release, which coordinates inhibition of food intake and increases energy expenditure via hypothalamic Lep receptor (Lep-R) activation.^(10,13)^ Thus, over the long term, the activation of Lep-R regulates body weight and adiposity.^(13)^ In addition, signs of short-term satiation are produced by intestinal cells, particularly the glucagon-like peptide-1 (GLP1) hormone produced by L-intestinal cells. GLP1 is secreted mainly by gut L-cells in response to fat and carbohydrate loading, but is also secreted by neurons in the central nervous system (CNS), especially in the brainstem. In response to peripheral and central actions, GLP1 acts on GLP1 receptors (GLP1-R), located in several hypothalamic areas, as a phasic regulator of satiety.^(13)^ Alterations in expressions and signaling of the Lep-R and GLP1-R in specific regions of the hypothalamus have been associated with obesity abnormalities.^(14)^

Aerobic exercises, particularly those maintained over the long term, can reduce WAT accumulation and preserve metabolic health, avoiding the development of diabetes and cardiovascular diseases in adulthood.^(15,16)^ Interestingly, regular exercise also modulates hypothalamic pathways, changing GLP1 and Lep responses, and potentiating the efficacy of GLP1-R agonist treatments in patients with Type 2 *diabetes mellitus*.^(17)^ Moreover, diabetic rats that are exercised demonstrate increased GLP1-R.^(17)^ Lep-R expression is also sensitive to central exercise effects. For example, 12 weeks of wheel exercise is reported to reduce the expression of Lep-R in the arcuate hypothalamic nuclei.^(18)^

Manipulating the litter size during lactation can modulate milk production and its components, provoking changes in the metabolism of offspring.^(19,20)^ Adult offspring born to small litters (SLs; 3 - 4 pups per dam) present obesity, dyslipidemia, and glucose intolerance,^(19,21,22)^ caused by lactation hypernutrition.^(5,23)^ Rodent dams feeding SL during lactation produce a milk that is richer in total calories, particularly lipids, compared to milk from dams with normal litters (NLs; 6–9 pups per dam). Accordingly, adults raised in SLs present alterations in hypothalamic pathways.^(24)^ SL-obese adult rats have been reported to have increased Lep-R expression in the hypothalamus^(25)^ and male SL-obese rats have exhibited reductions in hypothalamic GLP1-R.^(26)^ We and others have demonstrated that submitting SL rats to exercise can improve their metabolism and prevent the development of obesity.^(22,27)^ However, whether these anti-adiposity effects that are induced by exercise involve hypothalamic reorganization is unknown. Our hypothesis it is that regular exercise could restore hypothalamic pathways, contributing to positive health effects in overnourished SL adult male rats.

## OBJECTIVE

We evaluated the effects of swimming training on the expressions of hypothalamic genes GLP1-R and Lep-R in lactational hypernutrition-induced obesity.

## METHODS

### Ethical aspects and experimental design

**Figure 1:**
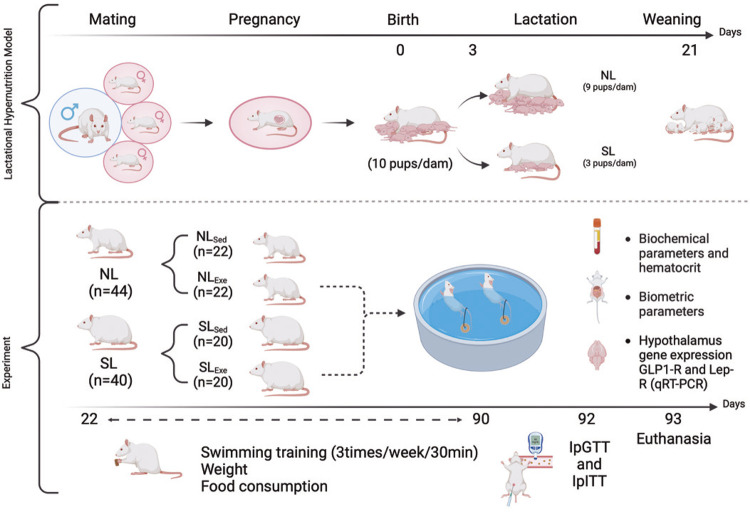
Experimental design

NL: normal litter; SL: small litter; NL_Sed_: normal litter and sedentary rats; NL_Exe_: normal litter and exercised rats; SL_Sed_: small litter sedentary rats; SL_Exe_: small litter exercised rats; IpGTT: intraperitoneal (ip) glucose tolerance test; IpITT: intraperitoneal (ip) insulin tolerance tests.

Wistar rats were obtained from the *Universidade Estadual de Ponta Grossa* (UEPG). The Ethics Committee for Experimental Animals (CEUA number 03482/2012) approved all animal protocols. Animals were kept under controlled temperature (21±3°C), humidity (50-65%) and luminosity (12:12h light-dark cycle), with *ad libitum* access to food and water. Male and females were matched, and pregnant female were separated until the birth of pups for litter size manipulation during the lactation phase. After weaning (21 days), rats were subdivided in sedentary and exercise groups. Exercised rats swam throughout their life. To avoid female hormonal influences, only males were used in the present work. The glucose and insulin tolerance test were performed *in vivo* (post-natal day 92), and the *ex vivo* analyses was performed after euthanasia (post-natal day 93). Details of each protocol are mentioned below and experimental design is shown in figure 1.

Illustrative experimental design created by author in Biorender.

### Induction of obesity by lactation hypernutrition

Wistar rats (age 90 ± 10 days) were mated at a ratio of three females to one male. After mating, pregnant females were placed in individual cages until the birth of offspring. Milk volume and composition of the dam can be adjusted to prole size.^(28,29)^ Therefore, at birth, the number of pups in each litter was kept to 10 per dams, to avoid maternal milk adjustment. On the 3^rd^ postnatal day, the lactating dams were randomly selected to have small litters (SLs; three pups/dams) or normal litters (NLs; nine pups/dams); only male rats were evaluated. Lactating dams suckling SL offspring produced milk with a high caloric content that was rich in lipids and carbohydrates, resulting in lactational overfeeding.^(21,30)^ SL (n= 40) and NL (n=44) offspring were weaned at 21 days of age and randomly distributed into 3 - 4 rats per cage, receiving water and rodent chow (Nuvital, Curitiba, Brazil) *ad libitum.* Dams and offspring were housed under a controlled luminosity cycle and temperature monitoring, as mentioned above.

### Swimming training

From 22 to 90 days of life, half of the NL (n=22) and half of the SL (n=20) male rats were submitted to swimming training, as described by Fischer et al.^(22)^ Briefly, rats swam 3 times/week for 30 minutes in a pool adapted to rodents (area 1m^2^), with constant temperature (32±2^o^C) and received a load corresponding to 5% of body weight attached to the tail, to avoid accommodation. This protocol can be considered to induce moderate intensity exercise, according to Voltarelli et al.^(31)^ After each swimming training session, the rats were dried and returned to their cages. Sedentary (Sed) groups did not perform any exercise. Importantly, all experimental protocols described below were performed at 48 hours after the last swimming session. Thus, considering litter size manipulation and swimming training, four subgroups were obtained (n=10-11 rats): NL_Sed_ - normal litter sedentary rats; NL_Exe_ - normal litter exercised rats; SL_Sed_ - small litter sedentary rats; SL_Exe_ - small litter exercised rats. All groups contained rats from at least five different litters.

### Feed efficiency coefficient

From 22 to 90 days of age, the animals were weighed and food consumption per rat determined (n=11 animals/group). The quantification of the feed efficiency coefficient (FEC) is the relationship between the body weight (BW) mass gain (g) per amount of feed consumed (g). The following calculation was used FEC = (final BW - initial BW) / Total of Food, as suggested by Nery et al.^(32)^

### Intraperitoneal glucose and insulin tolerance test

At 92 days of life, and at 48 hours after the last exercise session, the intraperitoneal (ip) glucose or insulin tolerance tests (ipGTT and ipITT, respectively) were performed; 8-10 male rats per group were randomly selected for each test. For this, the rats were allocated to individual cages and submitted to 12 hours (ipGTT) or 8 hours (ipITT) of fasting. Immediately before starting the test, a basal blood (time 0) sample was collected from the tail of each rat, and glucose levels were measured using a glucometer (Accuchek, Roche). Subsequently, the glucose load (2g/Kg of BW dose) was administered and glucose measured at 15, 30, 60, and 120 minutes after glucose administration. For the ipITT, after basal sample collection, the rats received insulin at a dose of 1U/Kg of BW and glucose was measured at 5, 10, 20 and 25 minutes after insulin administration. For both the ipGTT and ipITT, the areas under curve (AUCs) were calculated. Additionally, insulin sensitivity was analyzed by plasma glucose disappearance rate (K_ITT_), as reported by Lundbaek.^(33)^

### Euthanasia and blood and tissue collection

At 93 days of life, the male rats were fasted for 12 hours and euthanized by decapitation after brief desensitization with CO_2_. The rats were immediately weighed (g), and their naso-anal lengths (cm) were registered to obtain the Lee Index (LI).^(34)^ Subsequently, blood was collected in heparinized tubes and plasma was obtained was used to measure glucose, cholesterol and triglycerides using enzymatic commercial kits (Gold Analisa^®^, Belo Horizonte, MG, Brazil), and an automatic analyzer (Selectra II, Bayer), insulin was analyzed by radioimmunoassay. For hematocrit (Hct) evaluation, 1mL of blood was collected in tubes containing 1% EDTA-potassium, and hemoglobin (Hb; %), red blood cells (RBC), white blood cells (WBC) and platelet count, as well as the volume of concentrated cells, were determined. Additionally, the mean corpuscular volume (MCV), mean corpuscular hemoglobin (HCM), mean corpuscular hemoglobin concentration (MChC), monocytes (%) and lymphocytes (%) were evaluated in the blood. All hematocrit measurements were made in an automated cell counter (SERONO BAKER System 9120 CP + UK). After blood collection, the abdominal cavity of the rats was opened, and the WAT from visceral mesenteric and retroperitoneal depots and the brown adipose tissue (BAT) interscapular depot were excised and weighed; data are expressed in 100g BW.

### Quantitative real-time PCR (qRT-PCR) assay

Immediately after euthanasia, the brains of 7-8 animals/group were excised and weighed, and the hypothalamus was separated and transferred to TRIzol^®^ (Invitrogen, Life Technologies, Carlsbad, CA, USA) solution for RNA isolation, according to the manufacture’s protocol. For RNA expression, one microgram (µg) of total RNA was reverse transcribed using the First-Strand cDNA Synthesis Kit (GE Healthcare Bio-Sciences, Piscataway, NJ, USA), as recommended by the manufacturer′s instructions. For Lep-R and GLP1-R gene amplification, the following primer pairs were used, respectively: Lep-R_forward (5´CCATTCCCAGCTCACTGTCT 3´) and Lep-R_reverse (5´GAACAGGATTGAAACTGGGG 3´) and GLP1-R_forward (5´ CTGCATCGTGATAGCCAAGCT 3´) and GLP1-R_reverse (5´ GGACTTCGCGAGTCTGCATT 3´).^(35)^ The amplification of 18S rDNA (18S_forward: 5′ ATGCGTGCATTTATCAGA 3′; 18S_reverse: 5′ AACTATCCCGTCTGCAAG 3′) was used as an internal control.^(36)^ The qRT-PCR consisted of 20ng of template cDNA, 8μM of each primer (forward and reverse), and 1x SYBR Green PCR Master Mix Kit (Stratagene, La Jolla, CA, USA), in a total volume of 25μL. Gene expression was quantified by qRT-PCR in a thermocycler (Stratagene MxPro3005P, La Jolla, CA, USA) programmed as follows: 5 minutes at 94 °C; 40 cycles of 15 seconds at 94 °C, 30 seconds at 60 °C, 30 seconds at 72 °C; and ending with a dissociation curve. The threshold cycle (Ct) values obtained by amplification were measured and a relative change in the expression level of one specific gene was presented as 2 - ΔΔCt.^(37)^

### Statistical analysis

Data are presented as means ± standard deviation (SD). After normality and homoscedasticity tests, the data were submitted to two-way Anova and F values were calculated for Litter (L), Exercise (E) and Interaction (I), followed by the Tukey post-hoc test. P<0.05 was adopted as significant. Statistical analyses and figures were elaborated with GraphPad Prism, version 6.0 for Windows (GraphPad Software, San Diego, CA, USA).

## RESULTS

After weaning (21 days of life), the male SL rats presented higher BW (38%) in relation to the NL group rats (Table 1; p<0.0001); similar results were found in a SL adult male rat. Thus, at 93 days of life SL was found to influence BW (F _(1,40)_ = 32.07; p<0.0001), LI (F _(1,40)_ = 1.,76; p=0.0003) and fasting glycemia (F _(1,39)_ = 10.00; p=0.0030). SL_Sed_ rats presented higher BW (18%; p<0.0001); LI (4%; p=0.0040) and glycemia (46%; p=0.0060) in comparison to NL_Sed_ animals. Neither plasma triglyceride (F _(1,40)_ = 0.7940; p=0.3782) nor insulin (F _(1,38)_ = 1.58; p=0.2159) levels were significantly affected by litter manipulation (Table 1). With the exception of the insulin value, exercise influenced the BW (F _(1,40)_ = 10.42; p=0.0025), NAL (F _(1,40)_ = 8.375; p=0.0061), LI (F _(1,40)_ = 33.64; p<0.0001), and plasma glucose (F (_1,39)_ = 4.105; p=0.0496) and triglyceride (F _(1,40)_ = 4.165; p=0.0479) levels. Interaction effects (L *versus E*) were also observed for BW (F _(1,40)_ = 8.038; p=0.0071), where NL_Exe_ (16%; p<0.0001) and SL_Exe_ (11%; p=0.006) groups had lower BW in relation to SL_Sed_ rats. Moreover, LI was significantly reduced in NL_Exe_ (8%; p<0.0001) and SL_Exe_ (6%; p<0.0001) groups, in comparison to SL_Sed_ animals. Similarly, the fasting glycemia values were lower in NL_Exe_ (34%; p<0.0044) and SL_Exe_ (25%; p<0.0484) groups, in relation to the SL_Sed_ animals. Exercise and litter interaction effects modulated plasma triglyceride levels (F _(1,40)_ = 11.41; p=0.0016). Thus, SL_Sed_ animals demonstrated elevated triglyceride values, in relation to the NL_Sed_ (34%; p=0.0220) and SL_Exe_ (42%; 0.0024) groups.

**Table 1:** Fasting metabolic state, biometric parameters and adiposity in male adult NL and SL offspring submitted to swimming training

	NL_Sed_	NL_Exe_	SL_Sed_	SL_Exe_	p value		
					E	L	I
BW 21d (g)	33.11±7.15		45.56±4.25^£^				
BW 93d (g)	288.45±22.27^¶^	286.00±18.17^¶^	341.45±26.02^*,†,‡^	303.64±14.42^¶^	0.0025	<0.0001	0.0071
NAL (cm)	21.24±0.46	21.96±0.82	21.32±0.81	21.73±0.34	0.0061	0.7108	0.4322
LI	0.316±0.007^†, ¶^	0.304±0.011^*, ¶^	0.329±0.011^*,†,‡^	0.311±0.004^¶^	<0.0001	0.003	0.2407
Glucose (mg/dL)	68.36±10.45^¶^	66.50±20.60^¶^	100.55±29.99^*,†,‡^	75.82±20.28^¶^	0.0496	0.0030	0.0894
Triglycerides (mg/dL)	54.36±15.01^¶^	62.82±31.18	81.36±22.08^*,‡^	47.09±8.60^¶^	0.0479	0.3782	0.0016
Insulin (ng/mL)	0.23±0.25	0.22±0.12	0.31±0.29	0.47±0.74	0.5672	0.2159	0.5069

21d data was analysed by Student t test, ^£^ means p<0.05). Symbols above numbers indicate significance (p<0.05) in Tukey post-hoc test, *versus*: * NLSed; ^†^ NLExe; ^¶^ SLSed; ^‡^ SLExe.

Data are shown as means±SD (n=11 male rats/groups) and were submitted to two-way ANOVA with F effects presented in the p-value column (E: exercise; L: Litter; I: interaction).

BW: body weight; NAL: naso anal length; LI: Lee index; WAT- R: white adipose tissue retroperitoneal; WAT- P: white adipose tissue perigonadal.

**Figure 2:**
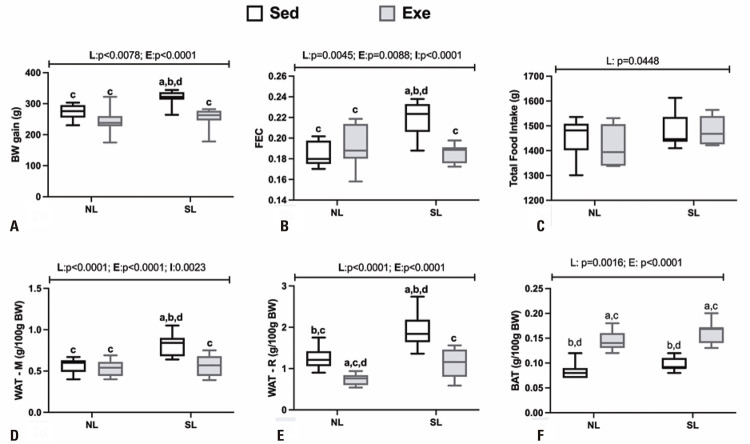
Body weight gain (A), FEC (B), total food intake (C) and adiposity (D to F) of NL and SL male adult offspring rats submitted to swimming training throughout life

The impacts of litter size reduction and swimming training on food intake, FEC and adiposity in adult SL offspring rats are shown in figure 2A-F. Litter size manipulation influenced BW gain (F _(1,40)_ = 7.842; p=0.0078; Figure 2A); FEC (F_(1,40)_ = 9.065; p=0.0045; Figure 2B); total food intake (F _(1,40)_ = 4.290; p=0.0448; Figure 2C); WAT-M (F_(1,40)_ = 22.35; p<0.0001; Figure 2D); WAT-R (F_(1,40)_ = 33.09; p<0.0001; Figure 2E) and BAT (F _(1, 40)_ = 7.536; p=0.0090; Figure 2F) content. Thus, SL_Sed_ rats showed higher BW gain (14%); FEC (22%) and visceral adiposity [WAT-R (47%) and WA-M (50%)] in relation to NL_Sed_ animals. Exercise also modified BW gain (F _(1, 40)_ = 20.63; p<0.0001); FEC (F _(1,40)_ = 7.596; p=0.0088); WAT-M (F _(1, 40)_ = 22.35; p<0.0001); WAT-R (F _(1, 40)_ = 50.91; p<0.0001) and BAT (F _(1, 38)_ = 140.7; p<0.0001). Moreover, interaction effects (L *versus* E) were noted in the FEC (F _(1,40)_ = 21.09; p<0.0001) and WAT-M depot (F _(1,40)_ = 10.62; p=0.0023). Thus, it may be noted that SL_Exe_ rats presented a lower BW gain (19%), and lower FEC (14%), WAT- R (41%) and WAT-M (31%), compared to the SL_Sed_ group, and showed similar values to those of the NL_Sed_ animals. Moreover, the SL_Exe_ and NL_Exe_ groups showed higher BAT weight (67%) in relation to the respective sedentary groups.

Data are means±SD. Male rats (10 -11) were used in groups originated from at least 5 different litters. The line(s) above of graphic indicate p values for F effects (E: exercise; L: litter and I: interaction) in two-way Anova test. Letters above bars show statistical differences, Tukey post-hoc test (p<0. 05) for; a: NL_Sed_; b: NL_Exe_; c: SL_Sed_; d: SL_Exe_.

**Figure 3:**
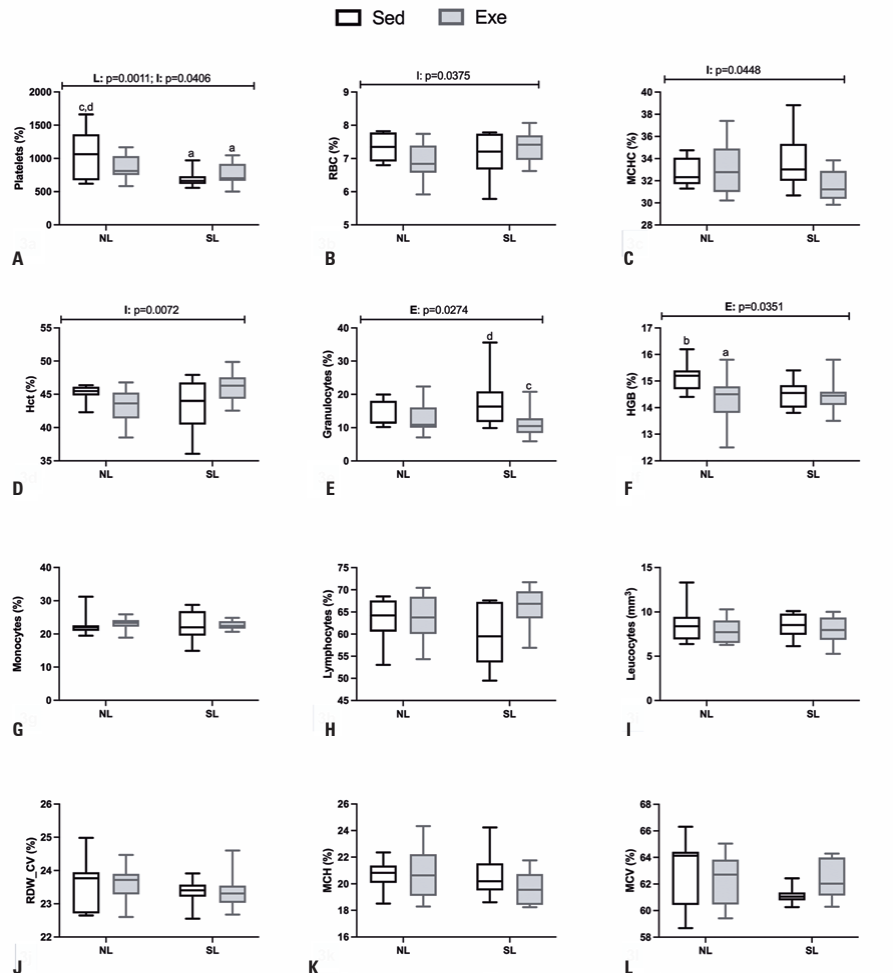
Red blood cells and white blood cells counts in adult normal litters and small litter male offspring rats submitted to swimming training throughout life

Blood cell parameters in the SL and NL exercised animals are presented in the figure 3A-I. The platelets were influenced by litter (F _(1,38)_ = 12.58; p=0.0011) as well as by the interaction (L *versus* E) (F _(1,38)_ = 4.495; p=0.0406). Thus, SL_Sed_ and SL_Exe_ groups presented significant reductions of 37% (p=0.0015) and 30% (p=0.0119), respectively, in platelet values, in comparison to NL_Sed_ animals (Figure 3A). Interaction effects (L *versus* E) were also observed for the RBC (Figure 3B), MCHC (Figure 3C) and Hct (Figure 3D) parameters, however without significant difference using the Tukey post-hoc test. On the other hand, exercise affected granulocytes (F_(1,38)_ = 5.263; p=0.0274; Figure 3E) and Hb (F _(1,38)_ = 4.773; p=0.0351; Figure 3F) values, where SL_Exe_ rats had a reduction of 36% (p=0.0492) in granulocytes in relation to SL_Sed_ animals. Moreover, NL_Exe_ animals had lower (5%; p=0.0428) Hb values in comparison to NL_Sed_ rats. The other hematological parameters evaluated were not significantly affected by either litter size manipulation or swimming training (Figure 3G-I).

Data are means±SD. Data are means±SD. Male rats (10 -11) were used in groups originated from at least 5 different litters. The line(s) above of graphic indicate p values for F effects (E: exercise; L: litter and I: interaction) in two-way Anova test. Letters above bars show statistical differences, Tukey post-hoc test (p<0. 05) for; a: NL_Sed_; b: NL_Exe_; c: SL_Sed_; d: SL_Exe_.

**Figure 4:**
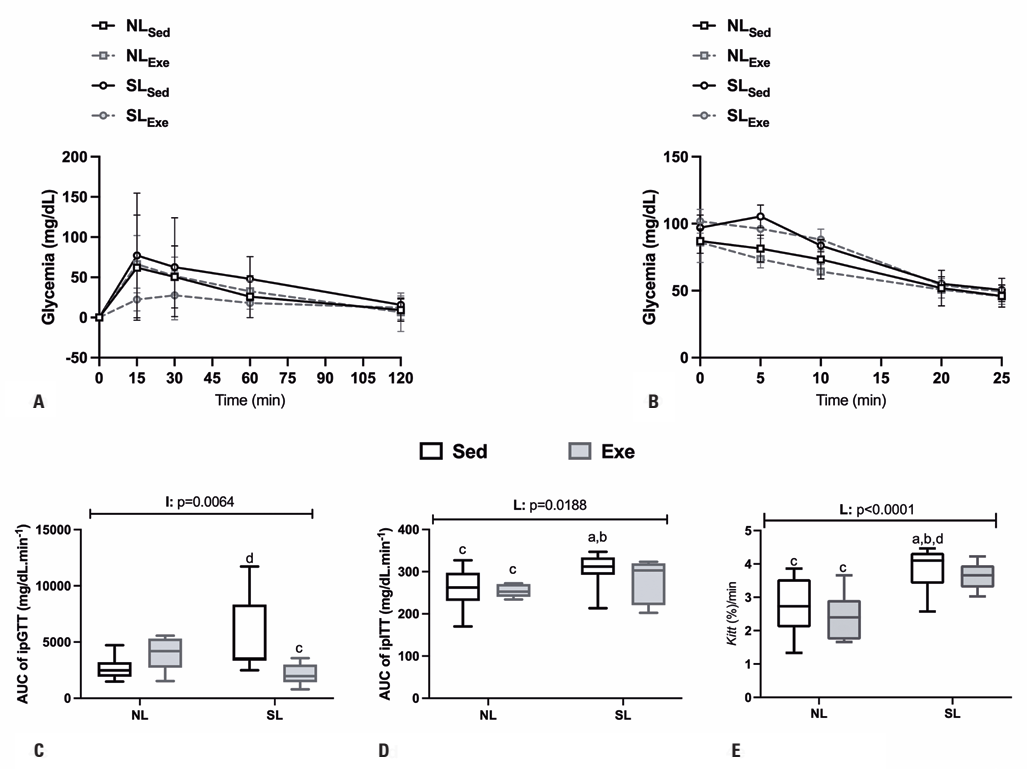
Glucose (A and C) and insulin (B and D) tolerance tests in normal litters and adult small litter male offspring rats submitted to swimming training throughout life

Glucose tolerance and insulin sensitivity are shown in figure 4A-E. After glucose load (2g/Kg/BW ip) the SL_Sed_ rats presented higher glucose values than those of the other experimental groups (Figure 4A), influencing AUC. The AUC of glucose during the ipGTT was affected by the E *versus* L interaction (F _(1, 27)_ = 8.734; p=0.0064), where SL_Exe_ rats presented a significant reduction (59%; p=0.0282) in the AUC of glucose, in comparison to the SL_Sed_ group (Figure 4C). The glycemic response after insulin load (1U/Kg/BW ip) was higher in the SL_Sed_ rats versus other experimental groups (Figure 4B), which influenced the AUC. The AUC was affected by the L interaction (F _(1, 31)_ = 6.149; p=0.0188) and SL_Sed_ rats had significantly higher glucose levels for the glucose AUC, in comparison to the NL_Sed_ (17%; p=0.0293) and NL_Exe_ (19%; p=0.0124) groups (Figure 4D).

Data are means±SD. Data are means±SD. Male rats (10 -11) were used in groups originated from at least 5 different litters. The line(s) above of graphic indicate p values for F effects (E: exercise; L: litter and I: interaction) in two-way Anova test. Letters above bars show statistical differences, Tukey post-hoc test (p<0. 05) for; a: NL_Sed_; b: NL_Exe_; c: SL_Sed_; d: SL_Exe_.

After insulin administration (1U/Kg/BW ip), plasma glucose levels decreased in all of the groups (Figure 4B). The AUC for glucose during the ipITT was significantly higher (17%) in SL_Sed_ animals, in relation to the NL_Sed_ and NL_Exe_ groups (Figure 4D). However, the magnitude of the decrease in blood glucose was greater for the SL groups, as shown by the K_ITT_ data (F _(1,31)_ = 25.47; p<0.0001; Figure 4E). Thus, SL_Sed_ rats showed an elevated K_ITT_ value in relation to NL_Sed_ (28%; p=0.0144) and NL_Exe_ rats (38%; p=0.0004).

**Figure 5:**
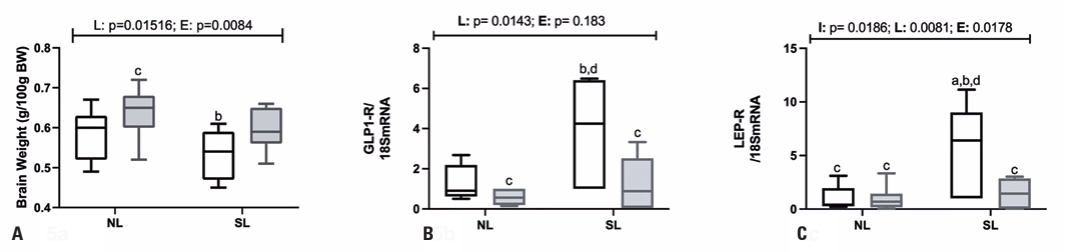
Total brain weight (A) and hypothalamic gene expressions of GLP1-R (B) and Lep-R (C) in adult normal litters and small litter male offspring rats submitted to swimming training throughout life

Total brain weight was influenced by litter size reduction (F _(1,40)_ = 6.381; p=0.0156) and exercise (F _(1,40)_ = 11.70; p=0.0015); total brain weight was significant lower (16%; p=0.0008) in the SL_Sed_ rats, compared to NL_Exe_ animals (Figure 5A). Similarly, hypothalamic GLP1-R gene expression was influenced by litter size reduction (F_(1,20)_ = 7.204; p=0.0143) and exercise (F _(1,20)_ = 6.607; p=0.0183; Figure 5B); hypothalamic GLP1-R gene expression was approximately five times higher in SL_Sed_ rats, compared to the NL_Exe_ (p=0.0096) and SL_Exe_ (p=0.0485) groups. Litter size reduction (F _(1,24)_ = 8.329; p=0.0081), exercise (F _(1,24)_ = 6.473; p=0.0178) and their interaction (L *versus* E) (F _(1,24)_ = 6.369; p=0.0186) influenced Lep-R hypothalamic gene expression; hypothalamic Lep-R gene expression was approximately five times higher in the hypothalami of SL_Sed_ rats, in relation to the NL_Sed_ (p=0.0064), NL_Exe_ (p=0.0062) and SL_Exe_ groups (p=0.0138) (Figure 5C).

Data are means±SD. Data are means±SD. Male rats (7-8) were used in groups originated from at least 5 different litters. The line(s) above of graphic indicate p values for F effects (E: exercise; L: litter and I: interaction) in two-way Anova test. Letters above bars show statistical differences , Tukey post-hoc test (p<0. 05) for; a: NL_Sed_; b: NL_Exe_; c: SL_Sed_; d: SL_Exe_.

## DISCUSSION

In the present study, we confirmed that pups reared in SLs present elevated BW at weaning and higher BW gain throughout life, as demonstrated by several studies.^(1,22,38)^ Moreover, our data show that adult SL offspring rats develop greater adiposity, as characterized by high visceral WAT depots, associated with augmented fasting values of glucose and triglycerides. These data corroborate results showing that lactational hypernutrition is associated with a high risk for obesity and metabolic diseases in adulthood, as established by the Developmental Origins of Health and Disease (DOHaD)concept.^(4,39)^ Lactational hypernutrition is a recognized model of obesity induction in adult life.^(5,21,40)^ We^(20)^ and others^(19,24,41)^ have demonstrated that SL reduction during lactation modifies milk composition, increasing lipid content and elevating the intake of calories by pups.^(4,19)^ High energy surplus during the lactation phase can alter important hypothalamic pathways involved in food intake and body weight control.^(4,5)^ In this regard, we found that adult SL offspring rats presented higher FEC, indicating a greater ability to convert calories consumed to BW gain, particularly visceral fat. These data confirm earlier findings that overnutrition in SL animals causes hypertrophy specifically in the visceral WAT depot.^(42)^ Some studies have indicated that adult SL offspring present elevated plasma insulin^(5,24)^ and leptin^(24,43)^ levels. In our study, we did not observe fasting hyperinsulinemia in adult SL offspring rats. However, we noted that these rats presented elevated Kitt values, indicating higher insulin sensibility. Insulin is a primary lipogenic and adipogenic hormone and probably contributed to elevated adiposity in the SL obesity model, as previously demonstrated by Lundbaek.^(33)^

As mentioned, adult SL offspring rats develop obesity and metabolic dysfunctions, such as glucose intolerance.^(22,40)^ We found that adult SL offspring rats had elevated AUC of glucose during ipGTT. However, this response was not linked to IR in adult life. We previously demonstrated that pancreatic islets from adult SL obese rats have reduced glucose-induced insulin secretion.^(22)^ Thus, the fasting hyperglycemia and glucose intolerance found in adult SL obese rats appears more related to the lower insulin secretion from the endocrine pancreas than IR, as also suggested by Waterland et al.^(40)^

Exercise elevates energy expenditure, reduces fat deposition, and prevents the development of chronic metabolic diseases.^(15,16)^ Accordingly, our data showed that swimming training, started after weaning and maintained throughout life, prevents obesity and metabolic abnormalities in adult SL offspring life. Herein, SL exercised rats demonstrated restored insulin sensitivity and glucose tolerance when compared to SL sedentary animals, events that may be related to normalization of fasting glycemia and the reduction in WAT visceral depots. Using the SL obese model, we previously demonstrated that swimming training is able to restore glucose-induced insulin secretion from isolated pancreatic islets.^(22)^ The anti-adiposity effects of exercise in SL rats were also demonstrated by Rinaldi et al.^(27)^ According to these authors, beneficial exercising of SL obese rats involves restoring the autonomic imbalance. Consistent with this finding, we also noted higher BAT weight in SL exercised rats, suggesting that swimming training was able to reactivate the BAT hypofunction frequently found in SL obese rats.^(43)^ In agreement, male rodents submitted to swimming training performed between 35-36^o^C showed augmented thermogenesis with high norepinephrine release and protein content, resulting in increased BAT weight.^(44)^

Obesity is associated with alterations in RBC and WBC numbers and functions.^(45)^ Herein, we showed that SL overfed rats presented normal profiles of RBC and Hct, in contrast to obese subjects.^(46)^ However, we observed a significant reduction in platelet count in SL obese rats, as is frequently observed in the obesity state.^(47)^ Activated platelets have thrombo-inflammatory functions linking hemostatic and immune responses in several physiological and pathological conditions.^(48)^ Early overnutrition elevates the risk for cardiovascular diseases during life.^(4,49)^ Thus, altered platelet counts in adult SL obese rats may contribute to abnormal vascular functions. Further studies are necessary to confirm this hypothesis. Moreover, we found interaction effects (E *versus* L) on Hct, RBC, MCHC and platelets, suggesting that the SL obese model could induce altered responsiveness of hematological parameters to exercise training.

In the present study, we also noted that gene expressions of GLP1-R and Lep-R are augmented in the hypothalamus of adult SL offspring rats, confirming several studies showing that lactation overfeeding modifies hypothalamic pathways and energy homeostasis control.^(2,50-52)^ Augmented Lep-R gene expression in hypothalamus was also found in adult SL obese rats by Aréchiga-Ceballos et al.^(53)^In contrast, Rocio Schumacher et al. showed that hyperexpression of the Lep-R gene observed in the hypothalamus of SL rats occurred only at 21 days, and disappeared in adult life.^(54)^ In contrast to Schumacher et al.^(54)^ who evaluated specific gene expression in the ARC, we studied gene expression in the total hypothalamus. Dawidowa et al. demonstrated that altered leptin anorexigenic responsiveness found in hypothalamic neurons of SL rats explained the disruption in energy homeostasis in this model.^(55)^ GLP1 is an important incretin that regulates food intake and insulin sensitivity;^(50,56)^ however, the effects of GLP1 have been poorly explored in the SL obese model. Our study found augmented GLP1-R hypothalamic gene expression in adult SL offspring rats. In contrast, a recent published study showed that adult SL rats present reduced GLP1-R gene expression in the ARC, without changes in intestinal GLP1-R protein expression.^(26)^ Similarly as Lep-R, GLP1-R gene expression was evaluated in the total hypothalamus and not just in the ARC, this may be the point of antithesis.

It is well recognized that epigenetic mechanisms, such as, histone and DNA methylation or acetylation besides miRNA are involved in lactation metabolic programming.^(57-59)^In SL rats, the hypothalamic gene promoter of the main anorexigenic neurohormone, proopiomelanocortin (POMC), showed hypermethylation of CpG dinucleotides, blocking leptin regulatory effects. Obesity is associated with leptin resistance and, despite hyperleptinaemia, POMC expression lacked upregulation in SL obese rats.^(57)^ A recent study demonstrated that decreased methylation of the Lep-R promoter, H3K27, increased Lep-R mRNA levels in the hypothalamus.^(58)^

GLP1-R is highly expressed in hypothalamic neurons, leading to an overall reduction of appetite and energy intake. Reduced expression of GLP1-R in Type 2 *diabetes mellitus* patients might also be mediated by increased methylation of its promoter.^(59)^ In endocrine pancreas, GLP1-R gene expression is regulated by DNA methylation, a process unknown at hypothalamic levels. Lep-R is present in the hypothalamic neurons expressing GLP1 and the anorexic action of leptin involves GLP1 regulation.^(60)^ Thus, we suggest that the hyperexpression of Lep-R and GLP1-R, found herein, could be related.

To our knowledge, we have shown, for the first time, that chronic swimming training effectively normalizes GLP1-R and Lep-R gene expression in the hypothalamus of SL overnourished rats, indicating a potential role of exercise in preventing the reprogramming induced by lactational hypernutrition. Plasma insulin and leptin can cross the blood-brain barrier (BBB), repressing neuropeptide Y (NPY)/AgRP (orexigenic neuropeptides) and increasing POMC (anorexigenic neuropeptide) gene expression in the hypothalamic ARC.^(61,62)^ Regular exercise can decrease high caloric food intake, leading to an improvement in body weight, due to activation of POMC neurons.^(52)^ In contrast to our findings, nine weeks of endurance exercise reduced plasma leptin levels and increased Lep-R mRNA expression in the ARC of non-obese mice.^(60)^ As demonstrated herein, SL-overnourished rats are obese and present high Lep-R hypothalamic gene expression. Thus, it is probable that exercise training restores altered Lep-R gene hypothalamic expression in SL-obese male rats. Interestingly, we have already demonstrated that exercise in SL rats restores the effects of GLP1 on isolated pancreatic islets.^(22)^ Moreover, as mentioned, the anorexic action of leptin involves modulations in GLP1-R in the hypothalamic nucleus.^(63)^

Swimming training can modulate hypothalamic gene expression via epigenetic mechanisms.^(64)^ In this study, swimming training was introduced immediately after weaning and maintained throughout life, favoring epigenetic exercise-induced modifications. In accordance with this hypothesis, long-term exercise elevated the levels of methylation in the hippocampus and hypothalamus, promoting down-regulation of gene related with adiposity.^(65)^ Thus, we believe that exercise-induced normalization of GLP1-R and Lep-R hypothalamic gene expression (shown herein) may be associated with the reestablishment of FEC, more adequate BW and adiposity control in adult SL offspring rats.

We should point out some of the limitations of our data. First, we did not measure the serum concentrations of leptin or GLP1. Secondly, we evaluated total hypothalamic GLP1-R and Lep-R gene expression, and these receptors can be modulated in specific nuclei, as demonstrated by Sanz et al.^(63)^

## CONCLUSION

In conclusion, swimming training throughout life can prevent the obesity and metabolic abnormalities induced by lactational overfeeding, an effect that could involve normalization of GLP1-R and Lep-R gene expression in the hypothalamus region.
